# Conspecific odor cues induce different vocal responses in serrate-legged small treefrogs, but only in the absence of acoustic signals

**DOI:** 10.1186/s12983-021-00415-y

**Published:** 2021-06-08

**Authors:** Ke Deng, Ya Zhou, Qiao-Ling He, Bi-Cheng Zhu, Tong-Liang Wang, Ji-Chao Wang, Jian-Guo Cui

**Affiliations:** 1 CAS Key Laboratory of Mountain Ecological Restoration and Bioresource Utilization & Ecological Restoration and Biodiversity Conservation Key Laboratory of Sichuan Province, Chengdu Institute of Biology, Chinese Academy of Sciences, Chengdu, 610041 China; 2 University of Chinese Academy of Sciences, Beijing, 100049 China; 3 Ministry of Education Key Laboratory for Ecology of Tropical Islands; Key Laboratory of Tropical Animal and Plant Ecology of Hainan Province; College of Life Sciences, Hainan Normal University, Haikou, 571158 China

**Keywords:** Odor cues, Advertisement calls, *Kurixalus odontotarsus*, Calling strategies, Anurans

## Abstract

**Background:**

Signal detection is crucial to survival and successful reproduction, and animals often modify behavioral decisions based on information they obtained from the social context. Undeniably, the decision-making in male-male competition and female choice of anurans (frogs and toads) depends heavily on acoustic signals. However, increasing empirical evidence suggests that additional or alternative types of cue (e.g., visual, chemical, and vibratory) can be used to detect, discriminate and locate conspecifics in many anuran species. Nevertheless, few studies have investigated whether conspecific odor cues affect male’s calling behavior. In this study, we conducted an experiment to investigate whether and how different chemical cues (male odors, female odors, and stress odors) from conspecifics affect male’s calling strategies in serrate-legged small treefrogs (*Kurixalus odontotarsus*), and whether the combined chemical and acoustic stimuli have additive effects on calling behavior or not.

**Results:**

We found that compared with female odors, male *K. odontotarsus* reduced calling investment in response to male odors or stress odors, in the absence of rival’s advertisement calls. When odor stimuli and advertisement calls were presented simultaneously, however, there were no differences in the vocal response of focal males among odor groups.

**Conclusions:**

These results provide evidence that male treefrogs switch calling investment according to different odor cues from conspecifics, and further demonstrate that calling behavior can be affected by chemical cues in anuran species. Our study highlights the potential role of airborne chemical cues in sex identification and contributes to increase our understanding of anuran communication.

**Supplementary Information:**

The online version contains supplementary material available at 10.1186/s12983-021-00415-y.

## Background

Survival and successful reproduction require animals to make critical decisions amidst a complex and dynamic social context [1]; therefore, it is important to accurately obtain relevant information in a given environment. It is well known that acoustic signals play a vital role in male-male competition and female choice of anurans (frogs and toads) [2, 3]. However, many anuran species use additional or alternative signal modalities to detect, discriminate and locate conspecifics [4, 5]. This may facilitate the efficient detection [6–8] and increase female choice preference or male competitiveness [9–11]. Although chemical cues were generally considered to play a minor role in anuran reproduction in contrast to other cues (e.g., acoustic and visual) [2], some studies have suggested that chemical cues could also help an individual in finding and selecting mates [12]. Furthermore, recent studies have implied that chemical cues might be used for close-range species or sex identification in treefrogs [13, 14]. However, very little is known about the behavioral response of individuals to different chemical cues from conspecifics in treefrogs.

**Fig. 1:**
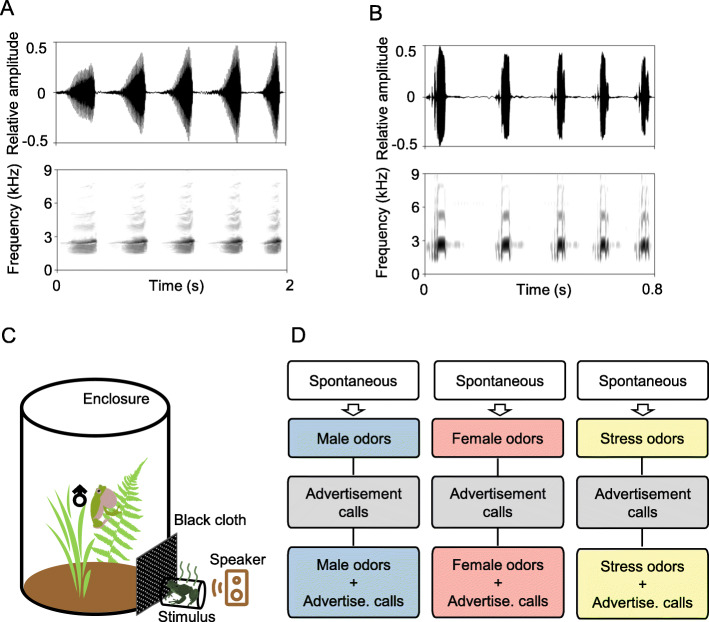
**a** Oscillogram (top) and spectrogram (bottom) of a typical advertisement call, which contains five A notes. **b** Oscillogram (top) and spectrogram (bottom) of a typical aggressive call, which contains five B notes. **c** Tested males were placed in cylindrical wire mesh enclosures (21 cm in diameter × 26 cm in height). An individual was placed in a wire cage (2 × 3 × 3 cm^3^ for males, and 2 × 4 × 5 cm^3^ for females) as an odor stimulus. Odor stimuli were positioned approximately 5 cm away from the enclosure, and a thin layer of black cloth (6 × 8 cm^2^) was placed between enclosure and cage to block visual cues. Acoustic stimuli were broadcast from 10 cm to the enclosure. **d** Tested males were assigned to a specific odor group. After the 3-min spontaneous period, odor stimuli, advertisement calls and the odor plus acoustic stimuli were presented in a randomized order with 3-min interstimulus intervals. Each stimulus period lasted for 3 mins

Serrate-legged small treefrogs (*Kurixalus odontotarsus*) are suitable model species to assess whether conspecific chemical cues affect male’s calling strategies. Generally, males vocalize on branches or in bushes to attract females. Males can produce two types of notes: a wideband A note and a narrowband B note [15]. Advertisement calls consist of a series of A notes (e.g., 5A, Fig. 1a), and it elicits vocal response and induces rivals to produce more aggressive calls [16]. In contrast, aggressive calls consist of a series of B notes (e.g., 5B, Fig. 1b), and it suppresses rival’s vocal response [16]. Our previous study demonstrated that male *K. odontotarsus* could detect potential mates through chemical cues, and adjust their calling strategies accordingly [17]. In addition, distinct stress odors can be detectable by human observers in this species (both sexes) when frogs are subjected to external threats, such as squeeze and rub (personal observations). Therefore, we conducted an experiment to investigate whether and how different chemical cues from conspecifics affect male’s calling behavior, and whether the combined chemical and acoustic stimuli have additive effects on calling behavior or not.

## Methods

### Study site

The study was conducted at Diaoluo Mountain National Nature Reserve in Hainan, China (18.72°N, 109.87°E, elevation 933 m). Our study was carried out May–August in 2020, during the reproductive period of *K. odontotarsus*. All experiments were conducted between 20:00 h and 01:00 h, and the average temperature was 22.15 ± 0.18 °C.

### Experimental design

Tested males were placed in cylindrical wire mesh enclosures (21 cm in diameter × 26 cm in height, Fig. 1c). The enclosures were placed in sites that were far enough (at least 30 m) from the chorus to prevent the focal males from directly interacting with other calling males. Soil and plants were provided, and the tested males could locomote freely in enclosures.

During the trials, tested males were allowed for a 3-min spontaneous calling period. Males who failed to vocalize within 15 mins after being placed in the enclosure were excluded from the study and were returned to the chorus. After the spontaneous period, odor stimuli, advertisement calls, and the combination of odor stimuli and calls were presented in a randomized order with 3-min interstimulus intervals (Fig. 1d). Each stimulus period lasted for 3 mins. Odor stimuli were positioned approximately 5 cm away from the enclosure, and a thin layer of black cloth (6 × 8 cm^2^) was placed between the enclosure and cage to block visual cues (Fig. 1c). During the interstimulus interval and the period of only advertisement calls were presented, the stimulus frog was removed by experimental executor. Acoustic stimuli were broadcast from 10 cm to the enclosure (Fig. 1c), and all stimuli were equalized for intensity (80 dB SPL, re 20 μPa), measured at the center of the enclosure using a sound pressure level meter (AWA 6291, Hangzhou Aihua Instruments Co., China).

To investigate whether male *K. odontotarsus* adjust calling strategies according to different chemical cues from conspecifics, tested males were assigned to one of the following three odor groups (Fig. 1d): male odors (normal odors from males, *N* = 47), female odors (normal odors from females, *N* = 38) and stress odors (*N* = 38). Normal odors were presented by an individual, which was placed in a wire cage (2 × 3 × 3 cm^3^ for males, and 2 × 4 × 5 cm^3^ for females). Stress odors were derived from a male that was handled gently by a mechanical arm (6 times per minute), which allowed him to release the distinct chemical cues.

All recordings were made with a digital voice recorder that was equipped with internal microphones (Sony PCM-D100). From the recordings, we counted the total number of calls, the total number of notes, the number of advertisement calls, the total number of A notes, the number of aggressive calls, and the total number of B notes during the spontaneous period and each stimulus period, which were obtained using Adobe Audition 3.0 software (California, USA).

### Statistical analysis

All data were square-root transformed (${x}^{\prime }=\sqrt{x}+\sqrt{x+1}$) to convert the zero values to the non-zero values [18]. The ratio of observed data in odor cues period to those in spontaneous period was used to evaluate vocal response to specific odor cue, and the ratio of observed data in odors plus calls period to those in advertisement calls period was used to evaluate vocal response to specific odor cue in the presence of acoustic signals. The original values were shown in the electronic supplementary material.

Data were examined using the Shapiro-Wilk test to determine their normality, and not all data indicated a normal distribution (*P* < 0.001). Consequently, to examine whether male *K. odontotarsus* adjust calling strategies according to different odor cues from conspecifics, and whether these effects still exist when presented with rival’s advertisement calls, we used the Kruskal-Wallis test to detect the differences among odor groups and used a two-sided Mann-Whitney U-test to determine the differences between pairs of groups. *P* < 0.05 was considered statistically significant. All statistical tests were performed using SPSS 20.0 (SPSS Inc., Chicago, IL, USA).

## Results

**Fig. 2:**
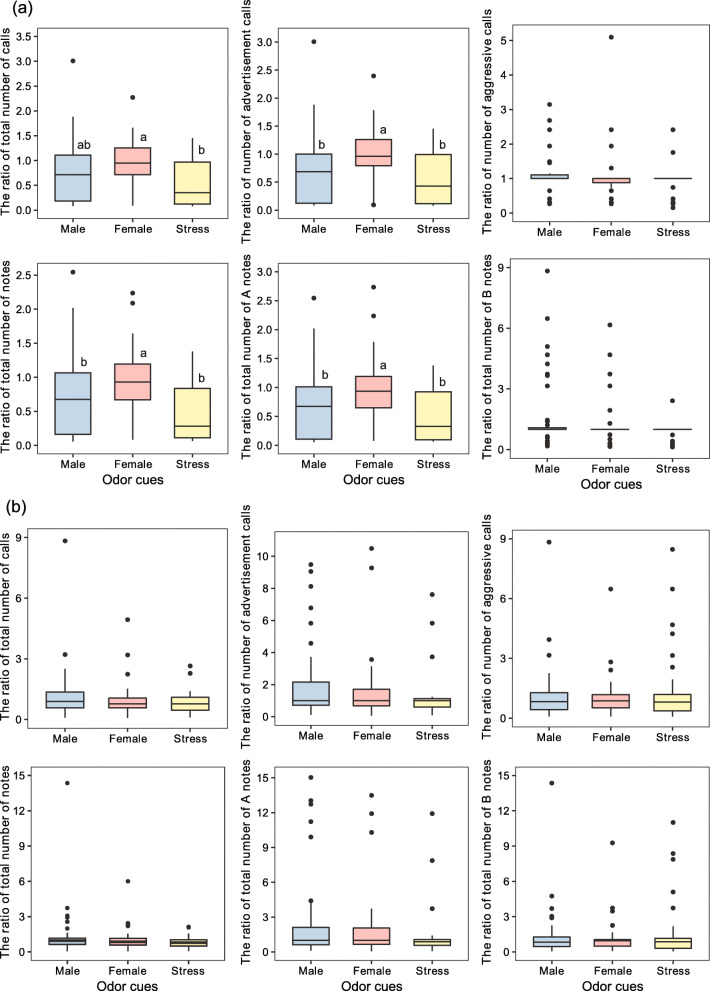
Comparison of different calls and notes in response to **a** only odor stimuli (the data in y-axis are the ratio of observed data in odor cues period to those in spontaneous period) and **b** odor stimuli paired with advertisement calls (the data in y-axis are the ratio of observed data in odors plus calls period to those in advertisement calls period). Male odors: *N* = 47; female odors: *N* = 38; stress odors: *N* = 38. Different superscript letters indicate significant differences (*P* < 0.05) as determined by the Mann-Whitney *U*-test

The ratios of total number of notes (*U* = 670.5, *P* = 0.049), number of advertisement calls (*U* = 602.5, *P* = 0.010) and total number of A notes (*U* = 620.5, *P* = 0.016) in male odor groups were lower than those in female odor groups (Fig. 2a). Similarly, the ratios of total number of calls (*U* = 423.0, *P* = 0.001), total number of notes (*U* = 414.0, *P* = 0.001), number of advertisement calls (*U* = 429.0, *P* = 0.002) and total number of A notes (*U* = 423.0, *P* = 0.002) in stress odor groups were lower than those in female odor groups (Fig. 2a). In contrast, the ratios of number of aggressive calls (Kruskal-Wallis test: *χ*^2^ = 2.938, *df* = 2, *P* = 0.230) and total number of B notes (Kruskal-Wallis test: *χ*^2^ = 2.829, *df* = 2, *P* = 0.243) did not vary significantly across odor groups (Fig. 2a). In the presence of both odors and advertisement calls, there were no significant differences in all measures among odor groups (Kruskal-Wallis test: all *P* > 0.05, Fig. 2b).

## Discussion

Our results show that male’s calling behavior is affected by different odor cues from conspecifics in *K. odontotarsus*, confirming the role of chemical cues in calling strategies in this species. Similar results have been found in a study of Australian terrestrial toadlet (*Pseudophryne bibronii*), which reported that the increase of calling rate of a focal male was more than twice as large in response to female odors compared with male odors, demonstrating that toadlets can use chemical cues to discriminate the sex of conspecifics [19]. In the present study, we found that compared with female odors, male *K. odontotarsus* reduced calling investment in response to male odors and stress odors. Generally, when rivals are known to be in close proximity, male frogs might stop producing advertisement calls, and switch to territorial calls or physical attacks [20–22]. On the other hand, we speculate that the stress odors of *K. odontotarsus* might be alarm cues. Studies across different taxa have indicated that when individuals perceive alarm cues from conspecifics, they generally reduce their activity [23, 24] or avoid the odor source [25–27]. For example, study with two neotropical treefrogs (genus *Boana*) suggested that both species interrupted their vocal activity and decreased call rate in response to conspecific distress calls, which might represent an alarm cue [28]. Therefore, it is a reasonable decision for male *K. odontotarsus* to produce relatively fewer calls when receiving odor cues that contain potential rivals or predation risks.

When odor stimuli and advertisement calls were presented simultaneously, we found no differences in the vocal response of focal males among odor groups, suggesting that acoustic signals predominate in male-male competition of *K. odontotarsus*. In recent years, increasing empirical evidence suggests that waterborne or airborne chemical cues can stimulate the behavioral response of conspecifics in anurans [19, 29–31]. For example, King et al. reported that a peptide isolated from norepinephrine-stimulated skin secretions from male mountain chicken frogs (*Leptodactylus fallax*) could elicit aggressive behavior in males [32]. Interestingly, this effect might be negligible [33] or even enhanced [34] when chemical cues are paired with conspecific calls, since different components either serve as back-up messages or provide different information [4]. Also, these inconsistent results suggest that behavioral response to acoustic plus chemical stimuli is species-specific in anurans, which has received less attention thus far, especially for airborne chemical cues.

In conclusion, we demonstrate that male *K. odontotarsus* adjust their calling strategies according to different chemical cues from conspecifics, but only in the absence of acoustic signals. These results imply that *K. odontotarsus* have the ability to discriminate different odors from conspecifics. Further studies are needed to examine the sex difference in volatile compounds, and determine the role of stress odors in chemical communication of *K. odontotarsus*.

## Supplementary Information

**Additional file 1.**

## Data Availability

Data used in this study are available in the electronic supplementary material.
